# Objective Assessment and Design Improvement of a Staring, Sparse Transducer Array by the Spatial Crosstalk Matrix for 3D Photoacoustic Tomography

**DOI:** 10.1371/journal.pone.0124759

**Published:** 2015-04-15

**Authors:** Philip Wong, Ivan Kosik, Avery Raess, Jeffrey J. L. Carson

**Affiliations:** 1 Imaging Program, Lawson Health Research Institute, St. Joseph’s Health Care, London, Ontario, Canada; 2 Department of Medical Biophysics, Schulich School of Medicine and Dentistry, Western University, London, Ontario, Canada; Shenzhen institutes of advanced technology, CHINA

## Abstract

Accurate reconstruction of 3D photoacoustic (PA) images requires detection of photoacoustic signals from many angles. Several groups have adopted staring ultrasound arrays, but assessment of array performance has been limited. We previously reported on a method to calibrate a 3D PA tomography (PAT) staring array system and analyze system performance using singular value decomposition (SVD). The developed SVD metric, however, was impractical for large system matrices, which are typical of 3D PAT problems. The present study consisted of two main objectives. The first objective aimed to introduce the crosstalk matrix concept to the field of PAT for system design. Figures-of-merit utilized in this study were root mean square error, peak signal-to-noise ratio, mean absolute error, and a three dimensional structural similarity index, which were derived between the normalized spatial crosstalk matrix and the identity matrix. The applicability of this approach for 3D PAT was validated by observing the response of the figures-of-merit in relation to well-understood PAT sampling characteristics (i.e. spatial and temporal sampling rate). The second objective aimed to utilize the figures-of-merit to characterize and improve the performance of a near-spherical staring array design. Transducer arrangement, array radius, and array angular coverage were the design parameters examined. We observed that the performance of a 129-element staring transducer array for 3D PAT could be improved by selection of optimal values of the design parameters. The results suggested that this formulation could be used to objectively characterize 3D PAT system performance and would enable the development of efficient strategies for system design optimization.

## Introduction

### Background

Photoacoustic tomography (PAT) is a hybrid imaging modality that combines the high spatial resolution of ultrasound (US) imaging with the high-contrast of optical imaging [1]. It is based on the detection of pressure waves generated by an object when exposed to laser light pulsed on the nanosecond timescale [2]. When an object is pulsed with laser light, a small fraction of energy is absorbed and emitted as a pressure wave due to the thermoelastic effect. The technique provides a means to estimate the optical properties of an object through ultrasonic detection of the pressure waves, which in turn provides information such as location, size, and composition of the object [3]. Moreover, since the optical properties between tissue types generally differ to a greater degree than their mechanical and elastic properties, PAT can provide improved tissue delineation and specificity when compared to imaging techniques that use US or photon propagation alone [4]. Objects such as human arm vasculature [5], whole-body small animal vasculature [6], human breast cancer [7,8], brain in small animals [9,10], and many others have been successfully visualized by PAT.

### PAT system design

The imaging performance for a given PAT system is heavily dependent on how well the photoacoustic (PA) signals generated from an object are detected. Signal fidelity is generally determined by the sensitivity and bandwidth of the transducer array as well as the number of projections captured [1]. Many PAT systems have been built where a single transducer, or an array of transducers, is mechanically scanned around an object to capture the data needed to reconstruct a 3D image. Although a large number of projections can be captured in this way, there is a trade-off between temporal resolution and image quality. Arising from the desire for fast real-time 3D PAT, several groups have begun to implement systems with staring transducer arrays [11–15], where image acquisition is limited only by the repetition rate of the laser. There has been much research on optimization of the temporal resolution for PAT systems that use mechanical scanning, but little emphasis on optimizing coverage for staring transducer arrays for a given transducer type. However, methods to systematically assess different designs are still lacking. Conventional metrics used to evaluate PAT system performance include sensitivity, resolution, and streak length, typically measured for a point source object [16]. These metrics, however, can be misleading in relation to PAT system design as they are typically measured after applying an image reconstruction algorithm to recover an image (usually a point source). Photoacoustic tomography systems that are designed with multiple imaging tasks in mind may be better optimized with generic figures of merit (FoM) that do not depend on the specific imaging task or the specific selection of image reconstruction algorithm used. In our earlier work, a higher level metric based on the singular value decomposition (SVD) was presented [11]. The metric derived from the SVD was based on examining the singular value spectrum. The number of effective singular values was proportional to the number of coefficients (i.e. voxels) recoverable, but comparison between two imaging systems would be problematic due to differences in singular vectors and their order. Each imaging system would have its own SVD and would be represented by a different set of singular vectors. Thus, the recovery of the same number of components for each imaging system is expected to produce images of different quality. Furthermore, calculating the singular value spectrum is computationally expensive and would be impractical for optimization tasks with larger system matrices, characteristic of 3D problems.

### Fourier crosstalk matrix

First introduced by Barrett and Gifford, the Fourier crosstalk matrix is a formulation shown to be an effective assessment technique for system design [17]. The Fourier crosstalk matrix provides insight into the contribution from each Fourier coefficient to the data as well as the amount of overlap between coefficients. Specifically, the diagonal elements of the matrix describe the strength of every Fourier coefficient in the data, being analogous to the generalized transfer function, while the off-diagonal elements represent the degree of linear dependency between any two Fourier coefficients [17]. The Fourier crosstalk matrix can be related to the Fisher information matrix, and hence, to task performance [18]. It has been successfully applied to characterize system design of cone-beam tomography [17], hybrid positron emission tomography (PET) [19], and computed tomography imaging spectrometry (CTIS) [20].

The crosstalk concept can be generalized to any basis functions that are appropriate for representing objects in image space. Reconstruction in PAT is inherently a shift-variant imaging problem and voxel (or pixel) basis functions are usually implemented instead of Fourier basis functions [15,21]. Voxel basis functions, however, are not localized in the frequency domain and may be ineffective in representing frequency-varying characteristics inherent to PAT. Our earlier work used the experimentally constructed spatial (or voxel) crosstalk matrix for a 15-element 3D PAT system [22]. We observed that the spatial crosstalk matrix qualitatively reflected changes in system performance as the transducer count and sampling rate were varied. Based on these qualitative findings, we were motivated to investigate quantitative measures derived from the spatial crosstalk matrix to quantitatively compare performance across different 3D PAT system designs.

### Objective and approach

Generic assessment techniques are a desirable alternative for optimization of PAT system design. Our previous work aimed to develop metrics for PAT system characterization based on the SVD, but these metrics were not well-suited for quantitative assessment of different system designs. The aims of this work were to explore more robust generic FoM for PAT system design assessment and to study methods to improve imaging performance of a staring array. The normalized spatial crosstalk matrix compared to the identity matrix was used to derive the FoM. The FoM considered were root mean square error (RMSE), peak signal-to-noise ratio (PSNR), mean absolute error (MAE), and a three dimensional structural similarity index (3D-SSIM). To our knowledge, this is the first time objective FoM from an experimentally constructed spatial crosstalk matrix have been applied to assess different 3D PAT system designs. The validity of the approach was tested by observing the response of the FoM as a function of transducer count and sampling rate. It is well understood in the field of PAT that system performance improves with increases in transducer count and sampling rate [2,11,23]. Photoacoustic targets often appear surrounded by streaking artifacts, which are reduced in intensity as the number of projections (transducers) is increased [24]. The Nyquist rate refers to the lower bound of the sampling rate required for alias-free signals and is defined as twice the highest frequency within the signal. Sampling below the Nyquist rate results in image artifacts, due to folding over (i.e. aliasing) of higher frequency content into lower frequencies. Sampling above the Nyquist rate allows for greater signal fidelity. Therefore, the FoM were expected to improve as transducer count and sampling rate increased. The effects of transducer arrangement, transducer array angular coverage, and transducer array radius on system performance were then estimated through a series of simulations and quantified using the FoM.

## Materials and Methods

### PAT system

**Fig 1:**
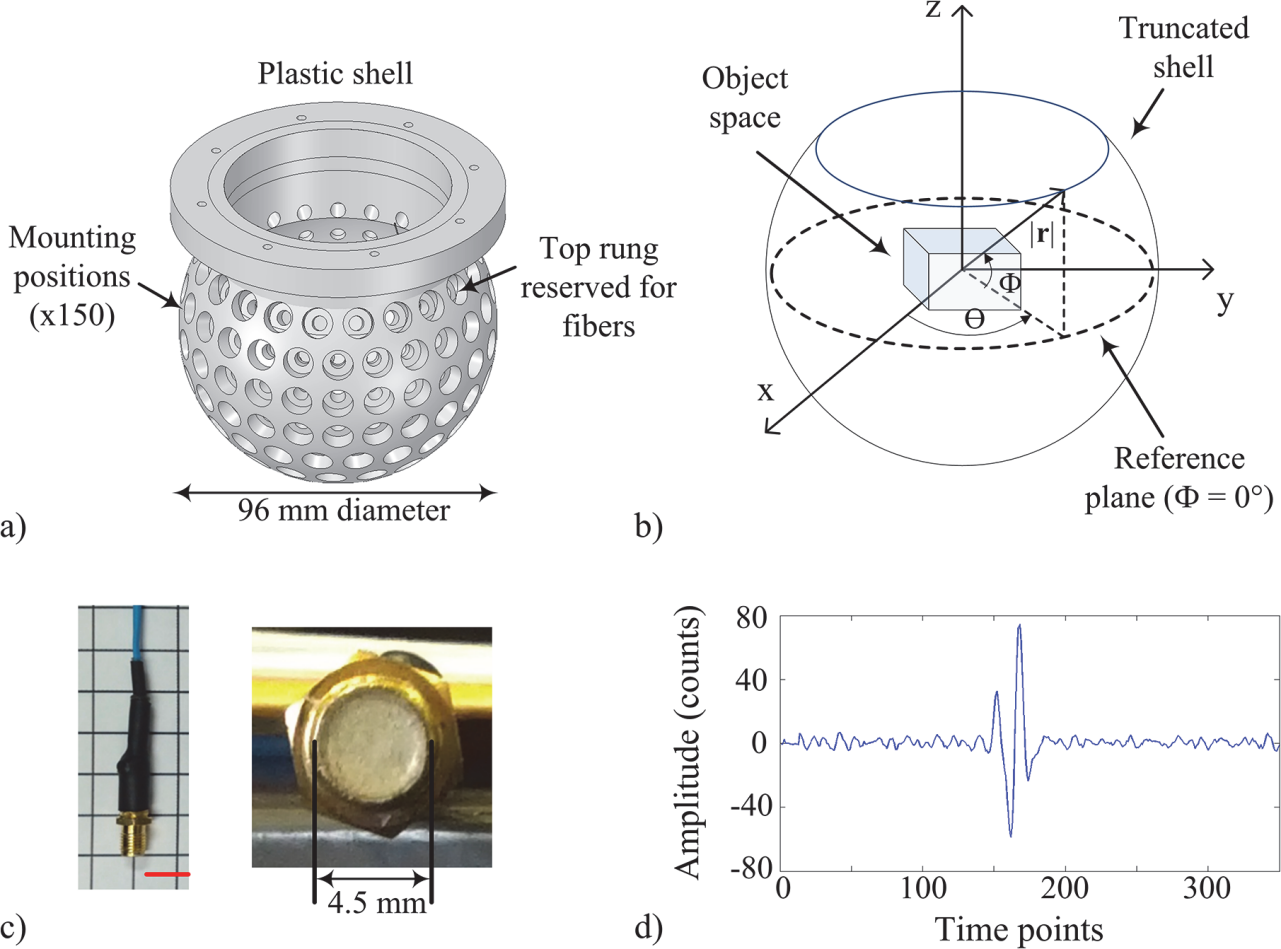
Staring transducer array design for 3D PAT. (a) A 3D CAD rendering of the plastic shell used to hold the transducers. (b) A 3D sketch of object space in relation to plastic shell. (c) Photograph of a custom-built transducer (left) and front face of the transducer (right). The red scale bar represents 1 cm. (d) Example of a photoacoustic pressure signal from a photoacoustic point source (~100 μm) averaged over 5 triggers acquired with one transducer at 40 MHz sampling rate. The amplitude represents the counts on the digital converter and ranges from ±2048 counts.

The 3D PAT system utilized a plastic shell (Fig 1(A)) designed to hold up to 150 transducers in a near-spherical staring arrangement. The inner surface of the shell had a radius of 48 mm. Transducer openings were located on 9 rungs evenly spaced along the zenith angle (Φ). The top rung was located at Φ = 30° (relative to a horizontal plane intersecting the center of the shell) (Fig 1(B)) and each consecutive rung were spaced in 15° increments. Mounting positions for the transducers were spread azimuthally (θ) counter-clockwise to provide uniform coverage over 360° within each rung, such that there were 21, 24, 24, 24, 21, 17, 12, 6, and 1 mounting positions in the top to bottom rung, respectively. The mounting position of the first transducer in each rung was longitudinally positioned at θ = 0°. A total of 96 custom-built cylindrical unfocused transducers (2.7 MHz central frequency, ~127% bandwidth, 4.5 mm diameter) were mounted into the shell at all but the top rung. All transducers were pointed toward the center of the shell. A photograph of the transducer is shown in Fig 1(C). The Nyquist rate for the transducers was estimated to be approximately 16 MHz according to the bandwidth of the transducer (i.e. transducers were able to detect up to ~8 MHz). The top rung was reserved for laser illumination (Surelite II Nd:YAG, Continuum, Santa Clara, California), which was delivered through two, four-legged optical fiber bundles that were inserted to provide diffuse illumination (4 x 3 mm x 1000 mm quartz fiber with fused input, Lumen Dynamics, Mississauga, ON). For these experiments, the fibers were not used as no imaging was performed. Data acquisition was performed with a 128-channel analog-to-digital data acquisition system (12 bit resolution, 40 MHz sampling rate, SonixDAQ, Ultrasonix Medical Corp., Richmond, BC) that was triggered on the electronic Q-switch signal from the laser. Data acquisition and data transfer were controlled through the SonixDAQ demonstration software and image reconstruction and image display were performed offline with a custom script for MATLAB (The Mathworks, Inc., version 7.8.0, Natick, Massachusetts).

### Spatial crosstalk matrix

The photoacoustic image reconstruction problem can be expressed as a linear continuous-to-discrete model:

g=Hf+e 1

where ***g*** represents the measured data set, ***H*** is the imaging operator (also referred elsewhere as the system matrix or forward model), ***f*** represents the unknown object(s), and ***e*** represents a noise process. The aim is to estimate ***f*** from the noisy data ***g*** but in most real situations the problem is ill-posed. Therefore, a linear regularization procedure is applied to minimize estimation error. A robust regularization procedure aims to minimize the aliasing (i.e. linear dependencies) in ***H*** without compromising signal intensity. Aliasing that is not removed during the inversion process may contribute to artifacts in the reconstructed images. Objective assessment of ***H***, prior to the regularization procedure, provides insight into system design and here we build upon our previous work and use the spatial crosstalk matrix. The spatial crosstalk matrix can be expressed as:

$$B=H^{T}H$$ 2

with elements defined by:

$$B_{jj\prime }=\sum_{k=1}^{K}(H_{jk}^{T}H_{j\prime k})$$ 3

where ***H*** ^*T*^ represents the transpose of ***H***, *j* and *j’* represent the index of the first and second voxel coefficient, *k* denotes the product of the time index for a given transducer and the index of the transducer, and *K* denotes the product of the total number time indices and the total number of transducers. Two important features can be derived from the spatial crosstalk matrix: system sensitivity and aliasing. The elements along the diagonal of the crosstalk matrix describe the system sensitivity, which represents the sum of the PA signal magnitude for all transducers at each voxel in object space. Examination of the off-diagonal elements of the crosstalk matrix provides a measure of aliasing at each voxel. Aliasing for a given voxel represents the amount of PA signal detected in surrounding voxels.

The spatial crosstalk matrix was computed according to Eq (2) for different ***H*** each constructed with a unique transducer count and temporal sampling rate. Each row of the spatial crosstalk matrix was then normalized to the value on the diagonal, creating a normalized spatial crosstalk matrix with ones along the diagonal. The transducer count was varied from 8 to 96 in increments of 8. Sampling rates of 5, 10, 20, and 40 MHz were used. The order in which mounting positions were defined around the bowl was used to guide transducer selection for a given transducer count. The mounting positions containing transducers were indexed from 1 to 96 counting counter-clockwise in each rung (from θ = 0°) starting with the second rung. For a transducer count of 8n, where n is the iteration index ranging from 1 to 12, transducers with indices 12m + 1 were selected, where m is 0, 1, …, n. This selection algorithm was used to provide even coverage over object space. Each row of the crosstalk matrix was normalized and reshaped into the spatial dimensions of object space. The FoM were calculated in reference to the correspondingly reshaped identity matrix.

### Calibration scan

The calibration scan involved experimentally measuring the PA time series (i.e. pressure modulations) due to PA sources in object space, thereby constructing ***H***. The calibration scan procedure has been described in previous work [22,25]. Briefly, light from a laser was guided through a 50-μm diameter optical fiber (Thorlabs, Newton, New Jersey). The output tip of the optical fiber was coated (Black Connector Coating, MG Chemicals, Burlington, Ontario). The coated tip acted as the photoacoustic point source and was mechanically fixed to the effector end of a SCARA robot arm (model E2C351S-UL with RC420 controller, Epson, Carson, California) to facilitate rapid and precise positioning. The input of the optical fiber was stripped of cladding and inserted into the 1064-nm beam of the laser. During calibration, the 4-legged optical fiber bundles were not used. Laser pulse energy was controlled by setting the Q-switch delay through software. A monitoring transducer (similar to one shown in Fig 1(B)) was attached to the robot arm at a fixed position relative to the point source in order to monitor changes in signal amplitude over the scan duration. The monitoring transducer signal was acquired with the SonixDAQ in parallel with the 96 transducer signals from the array and used to correct for variations in the array-based signals due to the variability of the laser energy from pulse to pulse. An example of an averaged signal from the monitoring transducer acquired over 5 triggers is shown in Fig 1(D). A scan volume of 20 x 20 x 20 mm^3^ centered with respect to the transducer array was sampled with a 500-μm grid spacing and 40-MHz temporal sampling rate (Fig 1(C)). The transducer signals due to each laser pulse were sampled for 25 μs (i.e. 1000 time points per transducer). The total size of the imaging operator was 96000 time points by 64000 grid points. The time to move the robot, fire the laser and capture the data took approximately 0.35 s per grid location and the entire scan took approximately 6 hours to complete.

The imaging operator was denoised using a filtering approach adapted from earlier work [22]. Previously, the peak size, peak width and time of flight were estimated from the acquired imaging operator data for each transducer and grid location and an inverted parabola was fitted according to these parameters. The denoised imaging operator in the current study was constructed by zeroing measured imaging operator data outside a defined window centered upon the time of flight for each transducer and grid location. A window size of 61 time points was determined empirically by examining the nominal width of the pressure signals across all grid points and transducers. Although the inverted parabola closely resembled the velocity potential of the bipolar pressure signal, it did not account for minor ringing associated with the measured response of each transducer. We chose not to use a previously developed liquid-based point source since the methylene blue absorber tended to stain surfaces such as the front face of the fiber tip thereby introducing systematic variability in the measured transducer signals over time [26]. Additionally, signal amplitudes from the liquid point source tended to be weak in comparison to the coated fiber tip.

### Figures-of-Merit

In the context of the spatial crosstalk matrix, the FoM utilized in this study are analogous to conventional image quality assessment (IQA) metrics. A common IQA image quality measure is root mean square error (RMSE) and is defined as

$$RMSE=\frac{1}{n}{\left(\sum_{t=1}^{n}{\left|e_{t}\right|}^{2}\right)}^{1/2}$$ 4

where *e*_*t*_ is the estimation error and *t* represents the index of the voxel elements in the image. Here we also utilized the IQA measures peak signal-to-noise ratio (PSNR) and mean absolute error (MAE) to provide additional system information [27]. They are defined as

$$PSNR=20log_{10}\left(\frac{MAX_{I}}{RMSE}\right)$$ 5

$$MAE=\frac{1}{n}\sum_{t=1}^{n}|e_{t}|$$ 6

where *MAX*_*I*_ is the maximum possible value within the image. These FoM are categorized as signal fidelity measures and are widely accepted due to their simplicity and clear physical meaning [27]. By measuring the differences between a test image to a reference image, *e*_*t*_ is obtained. In this case, the reference image was represented by the identity matrix (i.e. an image with no aliasing). The RMSE score will always have higher values than MAE and is more sensitive to large errors within the model [28]. The MAE is similar to RMSE, but has advantages over RMSE as an estimator of model performance [28]. The RMSE and MAE can also be analyzed together to provide additional information regarding error distribution [28]. Generally, if the difference between RMSE and MAE is small, the model tends to make many small errors, whereas if the difference is large, the model tends to make a smaller number of large errors. Both RMSE and MAE are negatively-oriented scores, whereas PSNR is a positively-oriented score.

In the field of IQA, it is well-known that signal fidelity measures do not match well with perceived visual quality (PVQ). Images with consistent RMSE or MAE values may have drastically different perceptual quality. Therefore, much effort has been placed in the development of PVQ metrics in order to approximate the human visual system, as it is the ultimate perceiver and appreciator of images. The structural similarity (SSIM) index is one of the most well cited PVQ metrics [29]. By comparing local patterns of pixel intensities, SSIM estimates image quality, or in this case system performance, as perceived changes in structural information [30]. The SSIM measure between two windows *x* and *y* of common size, where *x* is the windowed test image and *y* is the windowed reference image, can be expressed as:

$$SSIM\left(x,y\right)=\frac{\left(2\mu_{x}\mu_{y}+C_{1}\right)\left(2\sigma_{xy}+C_{2}\right)}{\left(\mu_{x}^{2}+\mu_{y}^{2}+C_{1}\right)\left(\sigma_{x}^{2}+\sigma_{y}^{2}+C_{2}\right)}$$ 7

where *μ* is the average, *σ* ^*2*^ is the variance, *σ* is the covariance and *C*_*1*_ and *C*_*2*_ are variables included to avoid instability when the denominator is very close to zero. We selected the default values used in [29] for *C*_*1*_ and *C*_*2*_. The window is displaced on the image and the calculation is repeated until the full image is covered. Averaging the repeated measurements gives the resulting SSIM value, where a value of 1 represents a perfect score (i.e. test and reference images are identical). The SSIM is not typically implemented on 3D images, but we adapted a 3D-SSIM method developed for video quality assessment (VQA), which was implemented on a data cube comprised of 2D images captured over time [31]. By simply extending *x* and *y* in Eq 7 into 3D windows of common size, we obtain the 3D-SSIM method. We used the 3D-SSIM method to analyze a data cube that represented 3D object space. 3D-SSIM The mean value across object space was calculated for each metric and was interpreted as measures of overall system performance.

### Simulations: 3D PAT system

The simulated 3D PAT system consisted of the same near-spherical shell of radius 48 mm and incorporated 129 transducers with sensing surfaces positioned tangentially to the surface of the shell. Transducers were spread across the bottom 8 rungs. For purposes of further discussion below, the transducer arrangement described above will be referred to as the *experimental transducer arrangement*. The transducer signal used in the simulations (Fig 1(A)) was the experimentally measured response of a custom-built unfocused ultrasound transducer to a PA point source [25]. Object space was defined as a cube 2 cm on edge located at the center of the transducer array comprised of grid points spaced 1 mm apart in a cubic lattice. For each grid point in object space, the transducer response was amplitude-scaled and time-shifted to account for the angle (with respect to the normal to the sensing element) and distance between the center of the transducer and the grid point. The amplitude-scaling values, *w*, were determined experimentally by modeling the transducer response as a function of angle and distance. The values were expressed as

$$w=\left(0.0325+0.768^{\alpha }\right)*2/d$$ 8

where α represents the angle between the grid point and the normal to the surface of the transducer face and *d* is the distance between the center of the transducer and the grid point. The speed of sound was assumed to be 1500 m/s for all simulations. All simulations were run in MATLAB (The Mathworks, Inc., version 7.8.0, Natick, Massachusetts).

### Simulations: system design improvements

System coverage (i.e. the aggregate transducer response) with respect to object space was expected to depend on the transducer arrangement, angular coverage of the transducer array, and radius of the transducer array. Therefore, parameter searches were performed through a series of simulations. For the first simulation, transducer arrangement was treated as a Tammes problem [32]. The Tammes problem refers to the placement of a given number of points, *N*, on the surface of a sphere such that the minimum distance between points is maximized [33]. In our case, the placement of points corresponded to 129 transducer positions (i.e. *N* = 129) and the Tammes problem was constrained to the surface of the truncated shell (i.e. Φ = 15°). The resulting transducer arrangement was referred to as the *uniform sampling arrangement*. The second simulation evaluated system performance as a function of angular coverage of the transducer array and was similar to the first simulation. Transducer arrangement solutions to the Tammes problem using 129 transducers constrained to Φ = 0°, 15°, 30°, 45°, and 60° for the truncated shell were tested. Note that Φ = 0° represented a hemispherical shell and Φ = 15° represented the shape of the *experimental transducer arrangement*. The third simulation evaluated system performance as a function of radius of the sensing surface. The radius was varied from 37.5 mm to 87.5 mm in increments of 10 mm. Transducers were positioned using solutions to the Tammes problem and the shell was fixed at Φ = 15°.

The spatial crosstalk matrix was computed according to Eq (2) for each system design and FoM were derived according to Eqs (4)–(7). Reference image maps were obtained by reshaping the corresponding rows of the identity matrix. The FoM were plotted as a function of concentric cubic shells in order to provide insight into the spatial-dependent response of each system design. The cubic shells were indexed from 1 to 10 according to their distance from the center of object space, where the innermost cubic shell was assigned the value 1 and the outermost cubic shell was assigned the value 10. The median for a given metric was calculated for the group of voxels located in a given cubic shell.

## Results

### System sensitivity and aliasing

**Fig 2:**
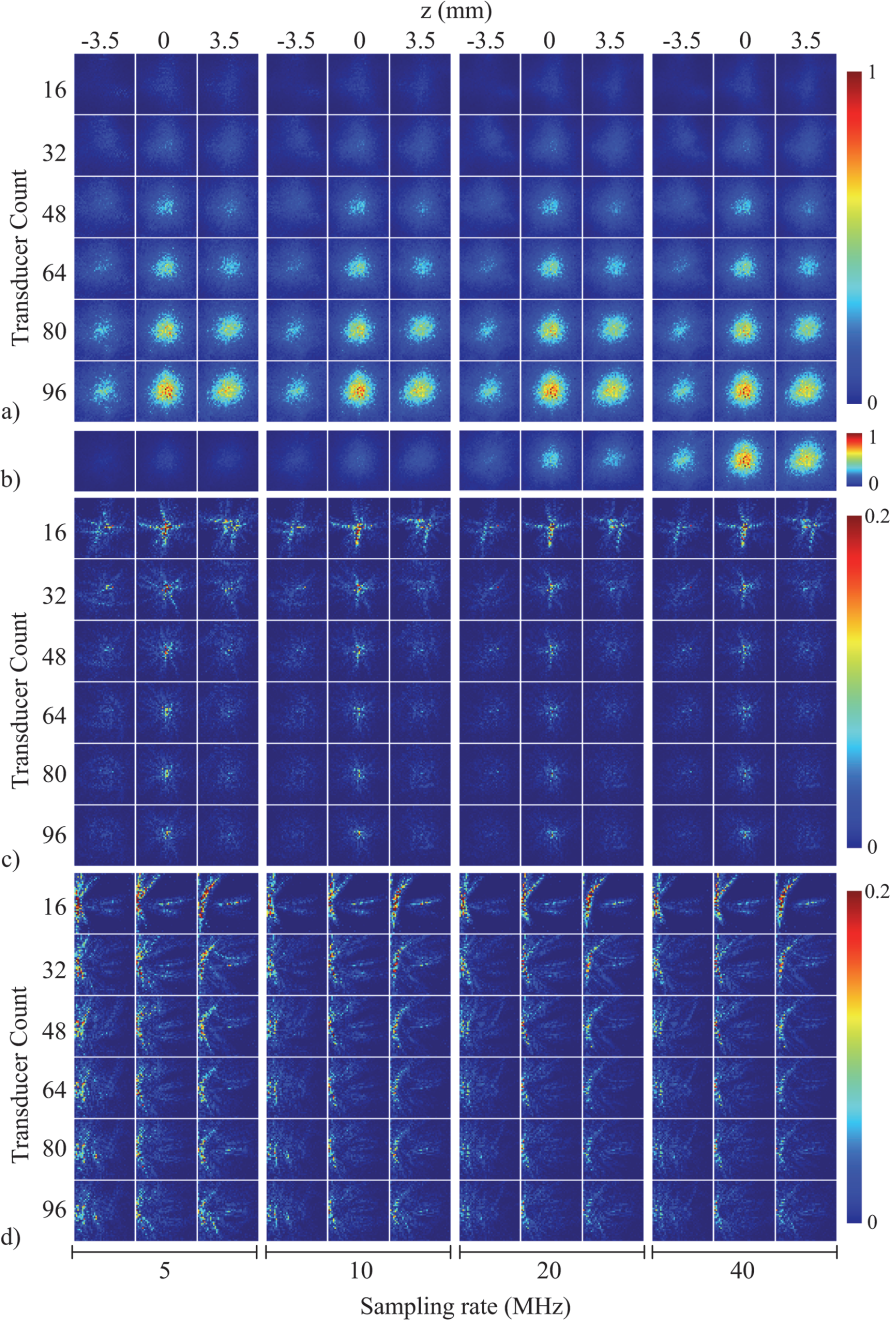
System sensitivity and aliasing as a function of transducer count and sampling rate. (a) System sensitivity maps of the xy-plane 3.5 mm below center (left column), center plane (middle column), and xy-plane 3.5 mm above center (right column) as a function of transducer count and sampling rate. Maps were normalized to peak system sensitivity per sampling rate. (b) System sensitivity maps showing last row of (a) (96 transducers) normalized to peak system sensitivity across sampling rates. (c) Aliasing maps for the center voxel as a function of transducer count and sampling rate. Planes correspond to those shown in (a). Maps were normalized to peak system sensitivity per sampling rate and per transducer count and then scaled to 20% max for display purposes. (d) Same as (c) but for aliasing for a voxel near the edge of object space.

System sensitivity, aliasing for the center voxel (i.e. near center grid point in the array), and aliasing for a voxel on the left edge of the scanned volume (i.e. grid point located 2 cm in the negative x-direction from the center grid point) are illustrated as image maps in Fig 2. The data in Fig 2 is presented as a function of transducer count and sampling rate. For each condition, three xy-planes near the center of object space (z = -3.5 mm, z = 0 mm, and z = 3.5 mm) are displayed. The system sensitivity maps in Fig 2(A) were independently normalized according to the peak value in each map. The system sensitivity maps in Fig 2(B) illustrate the last row of Fig 2(A) normalized to the peak value sensitivity across all maps. System sensitivity was spatially-dependent with a spherical shape. The region with highest system sensitivity occurred near the center of the truncated array and decreased with distance from the center. System sensitivity increased as transducer count and sampling rate increased (Fig 2(A) and 2(B)), with the highest sensitivity observed for the 96 transducers and 40 MHz sampling rate condition.

The aliasing maps displayed in Fig 2(C) and 2(D) were normalized to the peak value in each map in order to visualize changes in aliasing shape and pattern. At lower transducer counts and lower sampling rates, the aliasing pattern for the center and edge voxel appeared as streaks originating from the voxel position. As transducer count and sampling rate increased, streaking decreased. The trend was also true as a function of spatial position, where the center voxel had the least amount of streaking. The differences in aliasing at higher transducer counts and sampling rates were not easily distinguished from the image maps.

Aliasing structure appeared to be inversely proportional to system sensitivity, as observed when comparing aliasing from the center voxel (Fig 2(B)) to an edge voxel (Fig 2(C)). At regions in object space with lower system sensitivity, such as the edge, the aliasing map contained streaks. In regions with higher system sensitivity, the streaking was reduced and localized to nearby the voxel. Aliasing structure appeared to transition from streaking to blurring as transducer count and sampling rate increased. Sampling rates that did not meet the Nyquist criterion based on the transducer bandwidth resulted in aliasing maps corrupted with streaking patterns. However, artifacts in the system sensitivity maps computed for the lower sampling rates were not observably different from the system sensitivity map with sampling rate meeting the Nyquist criterion. Sampling above the Nyquist rate resulted in higher peak system sensitivity, but did not influence the aliasing maps substantially.

### Figures-of-merit

**Fig 3:**
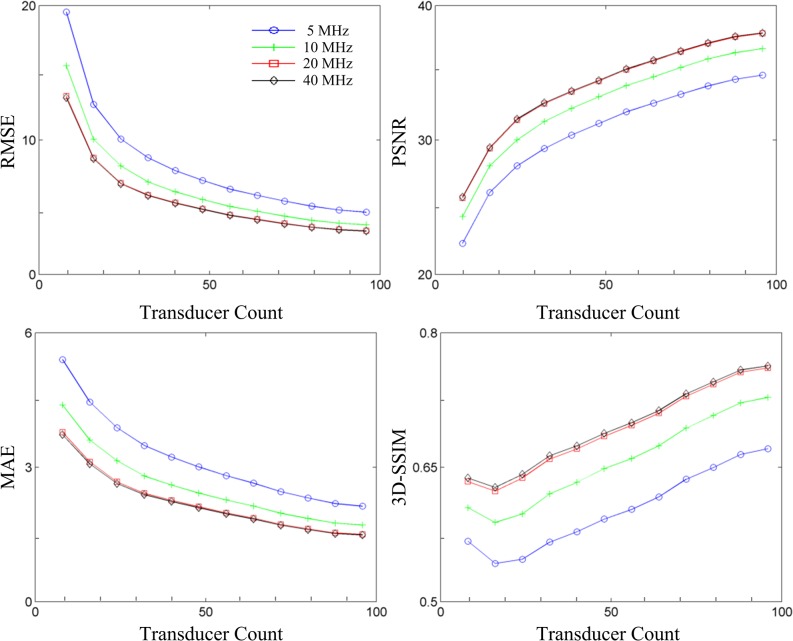
Figures-of-merit for system performance. Figures-of-merit (clockwise from top left: RMSE, PSNR, MAE, and 3D-SSIM) averaged over object space and plotted as a function of transducer count and sampling rate (legend shown in panel (a)).

**Fig 4:**
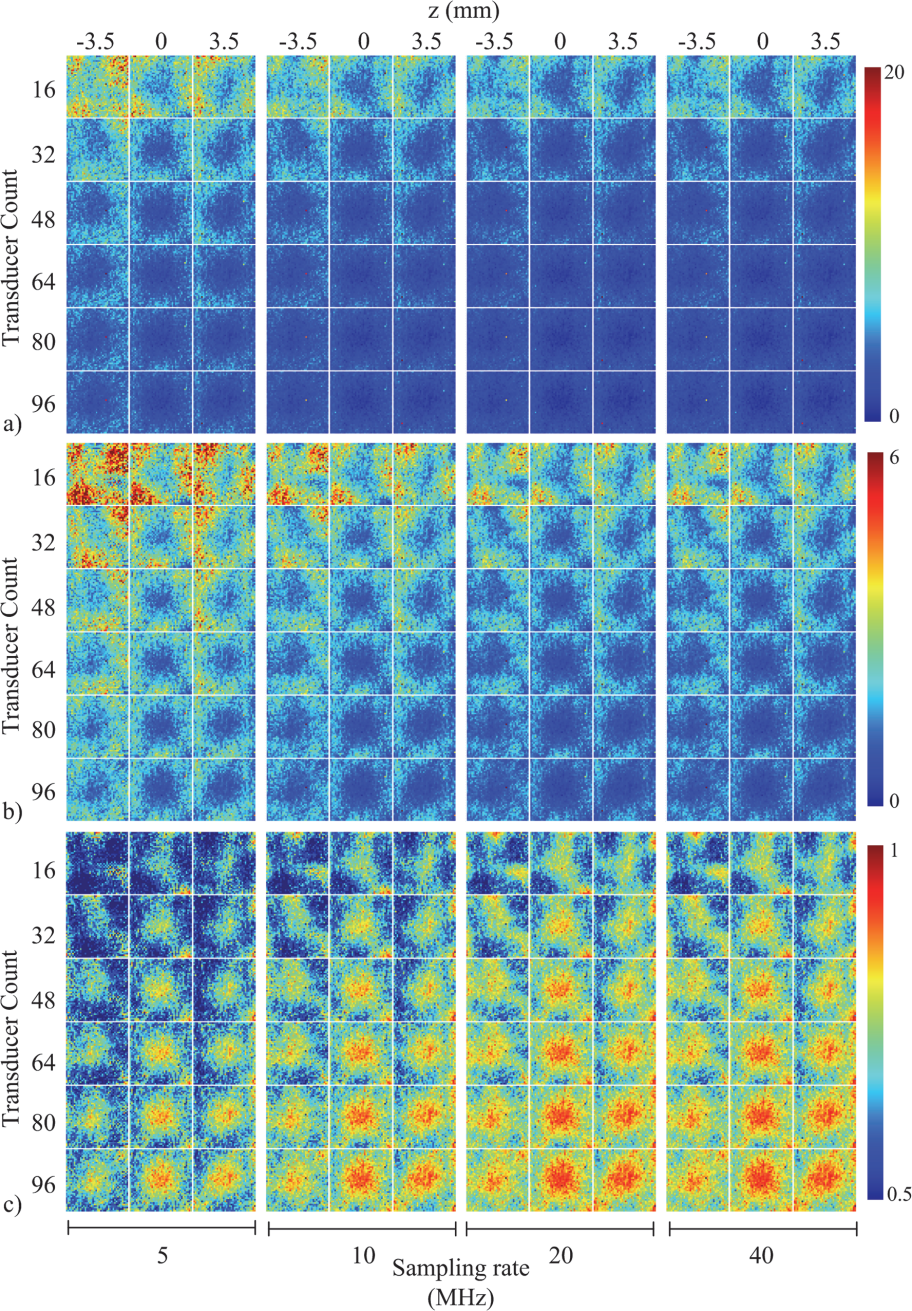
Metric maps for system performance as a function of transducer count and sampling rate. Metric maps for (a) RMSE, (b) MAE, and (c) 3D-SSIM displayed as a function of transducer count and sampling rate in the xy-plane 3.5 mm below center (left column), center plane (middle column), and xy-plane 3.5 mm above center (right column).

The responses of the FoM as a function of transducer count and sampling rate are plotted in Fig 3. The changes at higher transducer counts and sampling rates that were difficult to visualize in the aliasing maps in Fig 2 were quantified with the FoM. The FoM plots exhibited improved scores as transducer count increased. The curves in each FoM plot were similar in shape, but the curves corresponding to 5 MHz and 10 MHz exhibited poorer scores than the 20 MHz and 40 MHz curves. Specifically, the 5 MHz and 10 MHz curves were approximately 10% and 5% metric score units poorer than the 20 MHz and 40 MHz scores at each transducer count. The curves in the signal fidelity FoM plots appeared to be somewhat exponential in shape, whereas the curves in 3D-SSIM appeared to be substantially linear in shape. Furthermore, there was slightly less overlap between the 20 MHz and 40 MHz curves in the 3D-SSIM plot than in the other FoM plots. The spatially-dependent response of RMSE, MAE and 3D-SSIM as a function of transducer count and sampling rate are illustrated as metric maps in Fig 4. The layout of Fig 4 is similar to those in Fig 2, except for panel Fig 2(B). The shape of the metric maps matched well with the spatially-dependent system sensitivity and displayed best values in the center of object space. The FoM scores also appeared to improve in the three planes as transducer count and sampling rate increased.

### System design parameters: spatial crosstalk elements

**Fig 5:**
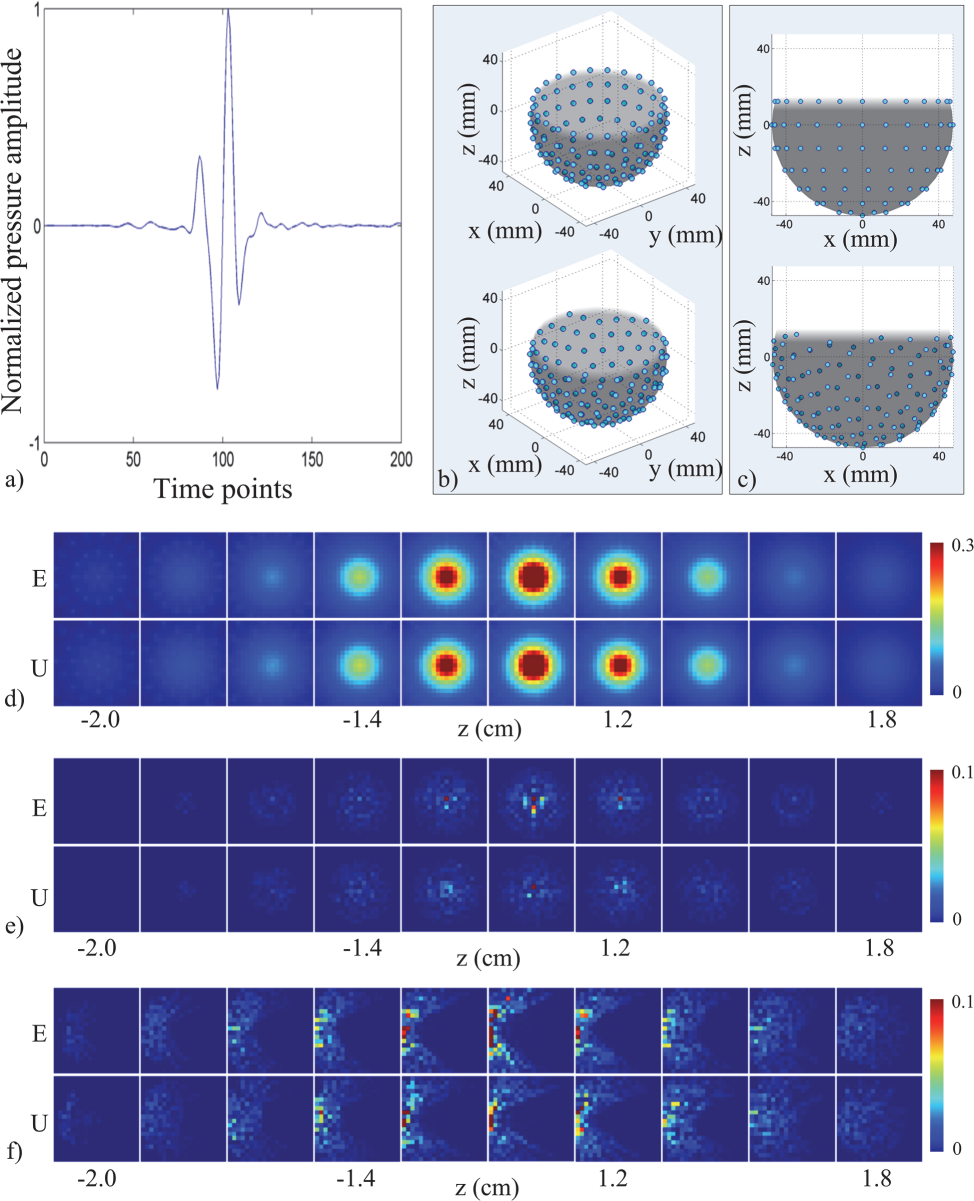
System sensitivity and aliasing for the *experimental transducer arrangement* and *uniform sampling arrangement*. (a) Transducer response profile to a PA point source (~100 μm) averaged over 64000 triggers acquired with one transducer at a 40 MHz sampling rate. The amplitude represents the counts on the digital-to-analog converter and has been normalized to the maximum sensitivity. (b) Point cloud representations of the *experimental transducer arrangement* (top) and *uniform sampling arrangement* (bottom). Darker shaded area represents exterior of shell closest to reader. Lighter shaded area represents interior surface of the shell. (c) Same as (b) from a side-view. (d) Normalized sensitivity maps for the two arrangements and scaled to 30% max sensitivity for display purposes. Aliasing maps for the center voxel (e) and a voxel along the left edge (f) shown for the two arrangements (*experimental transducer arrangement* in the top row and *uniform sampling arrangement* in the bottom row). Each arrangement was independently normalized and scaled to 10% max for display purposes. The image planes are 2 x 2 cm^2^ and correspond to every other xy-plane of object space (left to right corresponds to bottom (z = -2 cm) to top (z = 1.8 cm) planes at 2 mm step size).

**Fig 6:**
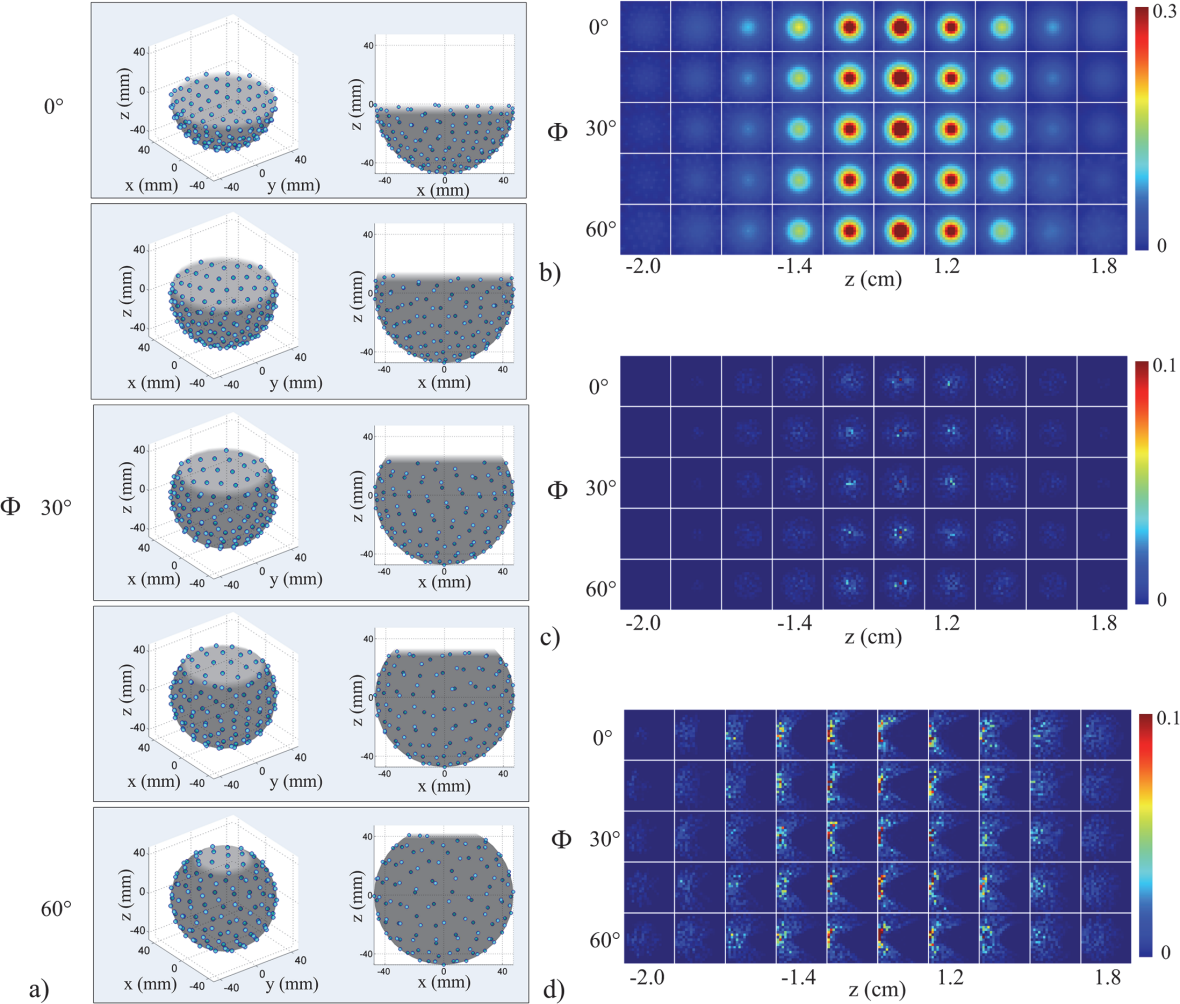
System sensitivity and aliasing as a function of array angular coverage. (a) Point cloud representations of transducer arrays as a function of array angular coverage (top to bottom corresponds to 0° to 60°). Side views are shown in the right-hand column. (b) Normalized sensitivity maps (scaled to 30% max for display purposes) for each array coverage angle (top to bottom row corresponds to 0° to 60°). (c) Independently normalized aliasing maps (scaled to 10% max for display purposes) for the center voxel and left edge voxel (d) for each array coverage angle. Image planes correspond with those in Fig 5.

**Fig 7:**
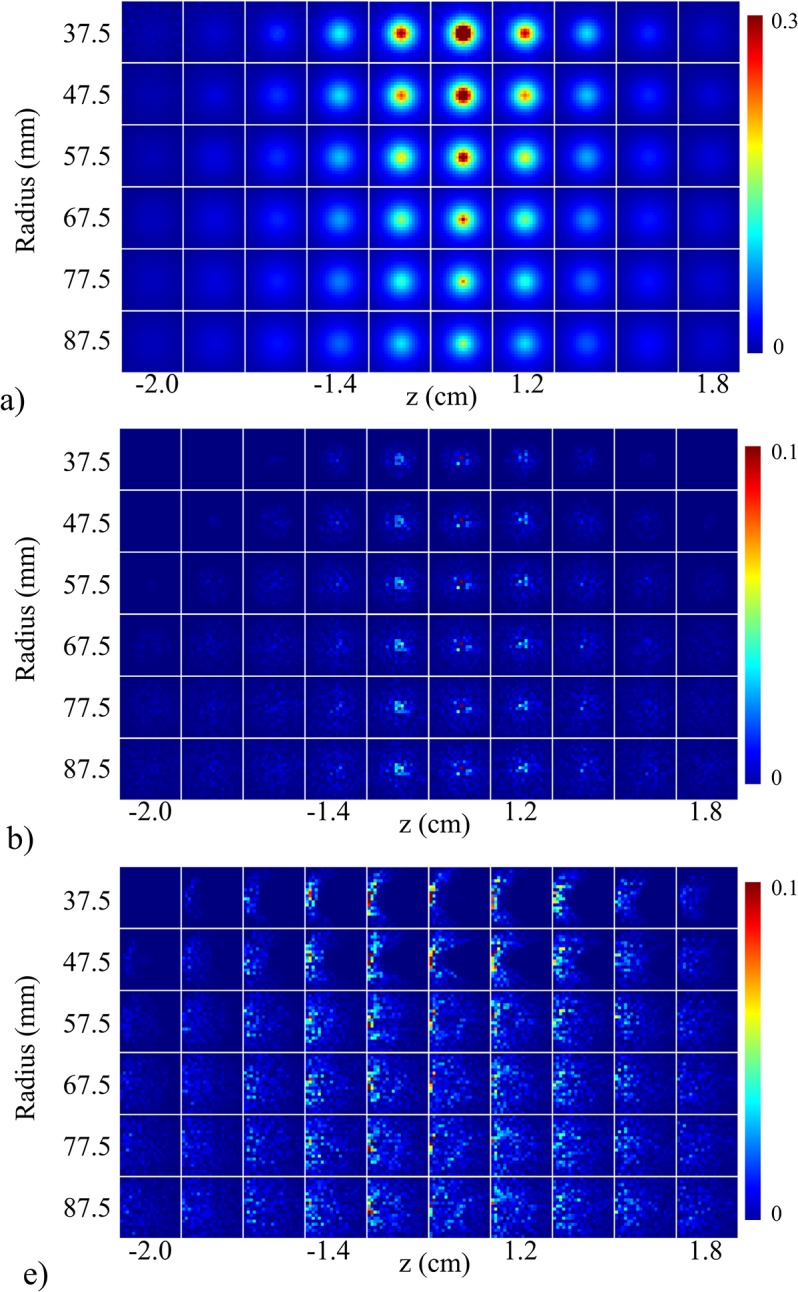
System sensitivity and aliasing as a function of array radius. (a) Normalized sensitivity maps (scaled to 30% max for display purposes) for each array radius (top to bottom corresponds to 37.5 mm to 87.5 mm, respectively, and Φ = 15°). (b) Independently normalized aliasing maps (scaled to 10% max for display purposes) for the center voxel and left edge voxel (c) for each array radius. Image planes correspond with those in Fig 5.

The experimental transducer arrangement and the *uniform sampling arrangement* are illustrated in the point cloud representations in Fig 5(B) and 5(C). From the point cloud representations in Fig 5(C), the pattern of transducers surrounding a given transducer appeared to be a rectangular lattice for the *experimental transducer arrangement* (E) and a hexagonal lattice for the *uniform sampling arrangement* (U). System sensitivity, aliasing for the center voxel (i.e. center grid point of the array), and aliasing for a voxel on the left edge (i.e. grid point located 2 cm from the center in the negative x-direction) are displayed as image maps for both transducer arrangements in Fig 5(D)–5(F). The image maps show equally-spaced xy-planes for object space, where image maps arranged left to right correspond to planes from the bottom to the top of object space. The system sensitivity maps in Fig 5(D) were normalized to the peak system sensitivity. The peak system sensitivity for each arrangement was observed at the center of object space, spherically-shaped, and spatially-dependent (decreased proportionally with distance from the center). System sensitivity appeared qualitatively similar for both arrangements, but, the *uniform sampling arrangement* appeared to have a more uniform system sensitivity response compared to the *experimental transducer arrangement*. The differences between the two transducer arrangements were more apparent in the aliasing maps (Fig 5(E) and 5(F)). Each aliasing map was independently normalized to the peak amplitude. The aliasing maps for the center and edge voxel appeared to have fewer artifacts for the *uniform sampling arrangement* compared to the *experimental transducer arrangement*, especially near the center of object space (z = 0 cm) Fig 6(A) display the point cloud representations of the system designs used to test angular coverage. The image maps in Fig 6(B)–6(D) correspond to the xy-planes displayed in Fig 5(D)–5(F). System sensitivity and aliasing maps were normalized according to the method used in Fig 5. Differences in system sensitivity and aliasing for the different cut-off angles (i.e. Φ) of coverage were small and hence not easily visualized in the figure panels, but system sensitivity in the lower and upper planes representative of object space appeared to differ slightly across the cut-off angles of coverage. For example, sensitivity in the top plane (z = 1.8 cm) appeared to be the smallest when Φ = 30° and largest when Φ = 60°. The point cloud representations and system sensitivity and aliasing maps for the system designs as a function of array radius are illustrated in Fig 7. System sensitivity and aliasing maps were normalized according to the method used in Fig 5. As array radius increased, system sensitivity decreased and aliasing increased. This was especially apparent near the center of the array. System sensitivity in the lower planes of object space, however, had greater spatial variation as array radius decreased.

### System design assessment: FoM

**Fig 8:**
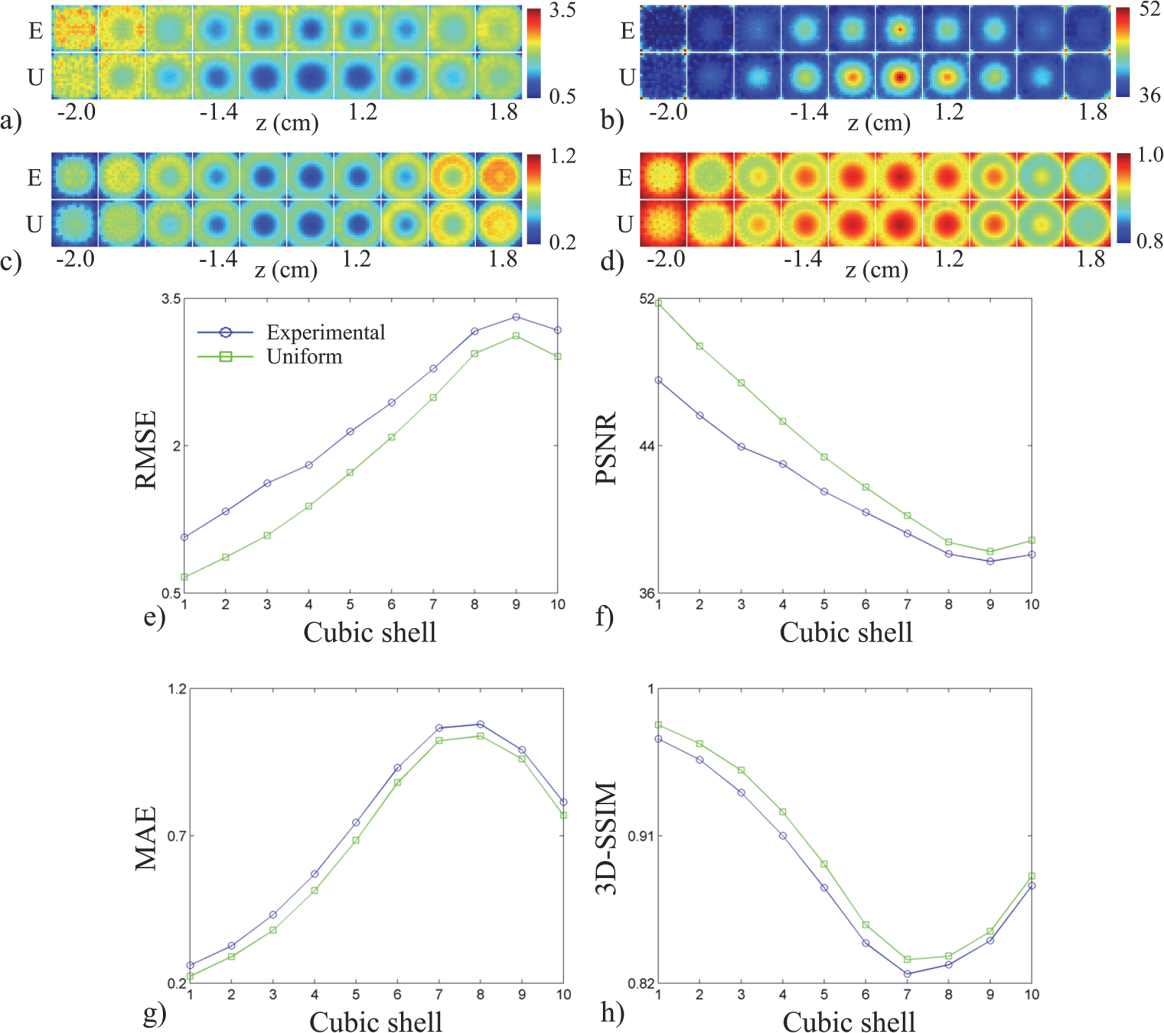
Effects of transducer arrangement on system performance. Metric maps for (a) RMSE, (b) PSNR, (c) MAE, and (d) 3D-SSIM displayed for both *experimental transducer arrangement* (top row) and *uniform sampling arrangement* (bottom row). (e)-(h) System performance figures of merit (RMSE, PSNR, MAE, and 3D-SSIM reading clockwise starting from top left panel) plotted as a function of cube contours for the two arrangements (legend shown in panel (e)).

**Fig 9:**
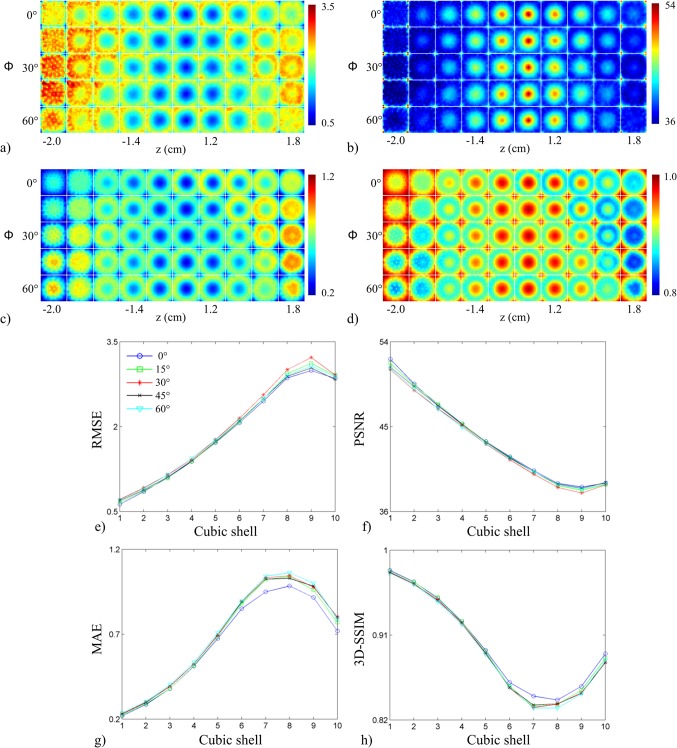
Effects of angular coverage on system performance. Metric maps for (a) RMSE, (b) PSNR, (c) MAE, and (d) 3D-SSIM displayed as a function of array angular coverage (top to bottom corresponds to 0° to 60°). (e)-(h) System performance figures of merit (RMSE, PSNR, MAE, and 3D-SSIM reading clockwise starting from top left panel) plotted as a function of cube contours with varying array angular coverage (legend shown in panel (e)).

**Fig 10:**
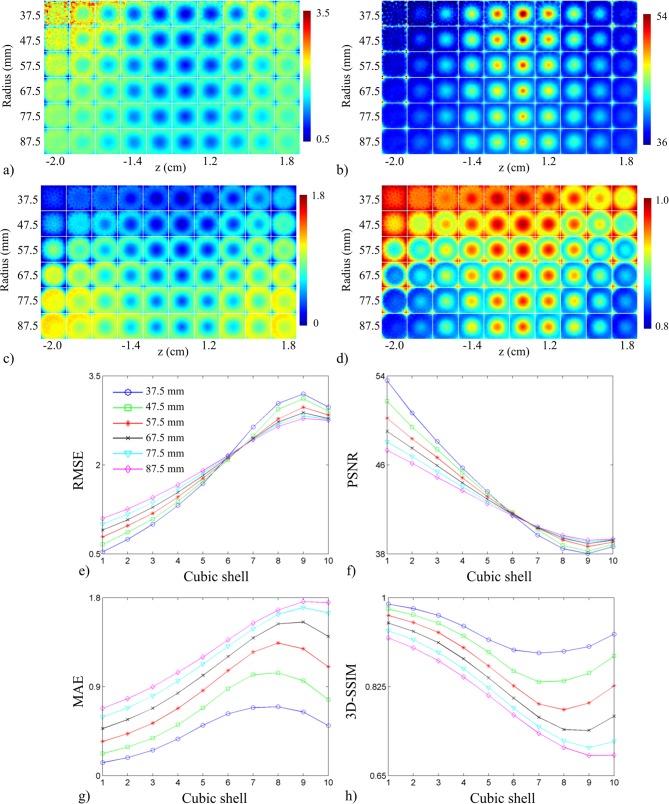
Effects of array radius on system performance. Metric maps for (a) RMSE, (b) PSNR, (c) MAE, and (d) 3D-SSIM displayed as a function of array radius (top to bottom corresponds to 37.5 mm to 87.5 mm). (e)-(h) System performance figures of merit (RMSE, PSNR, MAE, and 3D-SSIM reading clockwise starting from top left panel) plotted as a function of cube contours with varying array radius (legend shown in panel (e)).

The FoM for the first test are shown in Fig 8. The *uniform sampling arrangement* produced better scores than the *experimental transducer arrangement* in all FoM maps (Fig 8(A)), particularly in the center of object space. The distribution of the FoM maps for each arrangement was spherical in shape. Both arrangements exhibited similar curves when plotted as a function of concentric cubic shells (Fig 8(B)). The *uniform sampling arrangement* exhibited better scores than the *experimental transducer arrangement* at each concentric cubic shell. The FoM for the second test are shown in Fig 9. System response was almost identical across the different cut-off angles of coverage, but slight differences were apparent in the lower and upper planes of object space. As the cut-off coverage angles increased, system response showed poorer scores near the bottom and top of object space. The shape of the curves for all FoM was similar to those in Fig 8(B). Fig 10 displays the FoM for the third test. The FoM maps again appeared to be distributed with spherical symmetry. With decreasing array radius, voxel-to-voxel variation increased in the outer cubic shells. From the RMSE and PSNR plots, the curves intersected at the 6^th^ cubic shell, corresponding to an object space size of 1.2 cm^3^. The planes in the FoM maps shown in Figs 8, 9 and 10 correspond to those in Figs 5, 6 and 7.

## Discussion

### System sensitivity and aliasing

The PAT system we tested consisted of a 96-element near-spherical array with custom-built transducers. Improvements to the calibration process enabled acquisition of the system response with a finer resolution (500 μm) than our previous work (1.5 mm) and in a shorter period of time (~0.35 s per scan point versus 15 s per scan point). The improvement in resolution allowed for enhanced visualization and characterization of the system response. As described in Sec. 1.4, increasing spatial and temporal sampling rate was expected to result in improved system performance. We observed that the spatial crosstalk matrix was capable of qualitatively representing the improvements related to increased transducer count and higher sampling rate for the 3D PAT system (Fig 2). System sensitivity increased and aliasing was reduced as transducer count and sampling rate increased. An increase in the transducer count corresponded to an increase in the number of intersecting projections at a given point, which resulted in increased sensitivity. The higher number of intersecting projections for higher transducer counts also explained the transition of the aliasing structure from heavily streaked at low transducer counts to a blurred spot, as the number of streaking artifacts too large to visualize at higher transducer counts appeared as a blurred spot. Signal aliasing occurred when signals were sampled below the Nyquist rate, which resulted in a greater number of streaking artifacts in the aliasing maps. Sampling rates of 20 MHz and 40 MHz were above the Nyquist rate, which allowed for alias-free (frequency-based) signal detection. Therefore, system performance was nearly identical at the higher sampling rates. The spatial distribution of system sensitivity was spherically symmetric in shape with decreasing sensitivity with distance from the center (Fig 2(A)). This was expected from the near-spherical geometry of the array. Moreover, the directionality of the transducer faces, where each face was directed toward a common point at the center of the array, also contributed to the spherically-symmetric spatial distribution of system sensitivity. Since transducer sensitivity was lower for PA signals arriving off-axis to the normal of the transducer face than signals arriving straight on, the center of the array had the highest spatial sampling.

### Figures-of-merit

We have utilized generic FoM from the spatial crosstalk matrix for objective analysis of a 3D PAT system. The crosstalk elements displayed in Fig 2 were used to derive global FoM for system performance (Fig 3) as transducer count and sampling rate were manipulated. It is interesting to note that only 3D-SSIM required the rows of the crosstalk and identity matrix to be reshaped into the dimensions of object space. Near-voxel dependencies were incorporated by 3D-SSIM and then utilized to measure system performance according to the structure of the image. This may explain the difference in the shape of the curves between the signal fidelity FoM plots and the 3D-SSIM plot. It can be argued that aliasing (i.e. streaking artifacts) in PAT is better represented by the structural information of the image rather than the overall quality of the image. Therefore, 3D-SSIM would be more effective than signal fidelity FoM in detecting aliasing. The FoM were also visualized as metric maps (Fig 4) to examine local system performance. For both global and local system performance, the FoM aligned well with PAT sampling characteristics. As observed in Figs 3 and 4, the FoM scores improved with increasing spatial and temporal sampling rate. This suggested to us that the FoM derived from the spatial crosstalk matrix was an appropriate tool to characterize and optimize 3D PAT system design. The slight dip in the 3D-SSIM plot at the second point is likely due to the fact that the system matrix was experimentally calibrated, resulting in an inclusion of system noise and variable transducer performance that could degrade the overall perceptual quality of the aliasing maps. The global measure of system performance (i.e. the mean 3D-SSIM value) may also not have well-represented the true overall performance of the system.

### System coverage

With a fixed number of transducers, the transducer array coverage was examined by estimating the effects of transducer arrangement, array angular coverage, and array radius. The arrangement of transducers on the experimental system provided uniform coverage azimuthally within each rung but sub-optimal coverage along the zenith (top of Fig 5(C)). A more uniform distribution of transducers on the bowl was obtained by solving the Tammes problem (bottom of Fig 5(C)). The increase in coverage along the zenith accounted for the increase in uniformity of the system sensitivity (Fig 5(D)) as well as a decrease in aliasing (Fig 5(E) and 5(F)). System sensitivity and aliasing did not appear to change as a function of the cut-off angle of the array in the Φ direction. This was likely due to the fact that the number of projections acquired was still determined by the number of transducers and that the projections were uniformly distributed. System sensitivity decreased and aliasing increased as a function of array radius (Fig 7). System sensitivity also became more uniform with larger array radii. Positioning transducers farther away from the object space would increase the number of object grid points falling within the transducer acceptance angle, but reduce overall system sensitivity (Eq (8)).

### System design assessment

Deriving FoM from the spatial crosstalk matrix enabled an objective assessment of the sparse detection problem. Each spatial crosstalk matrix represented a different sparse detection design and was evaluated in reference to the identity matrix, representing a shift-invariant, full-view system. The off-diagonal elements of the crosstalk matrix represented the instability that arose from sparse detection and the FoM assessed how closely aligned a system design was to the ideal system with perfect sensitivity. The improved performance of the *uniform sampling arrangement* was quantified in Fig 8. The *uniform sampling arrangement* was shown to outperform the current transducer arrangement when compared across cubic shells in object space. The shape of the curves in the RMSE and PSNR plots indicated improved system performance proportional to the distance from the center of object space, where best system performance was observed at the center of the array. The MAE and 3D-SSIM plots showed a similar trend, except in the outer two cubic shells (cubic shell 9 and 10). The outer two cubic shells showed improved performance due to enhanced detection at the corners of object space (Fig 8(A)–8(D)). The corners of object space represented grid points that were closest to the transducers and hence, were well-detected. Fig 9 shows the results for varying array angular coverage. Similar to the system sensitivity maps in Fig 6, array angular coverage beyond Φ = 0° had little to no effect on system performance. Slight variations in system performance in the outer cubic shells of object space are highlighted in the FoM maps (Fig 9(A)–9(D)) and quantified in the FoM plots (Fig 9(E)–9(H)). The trends in the FoM plots matched the trends from the FoM plots in Fig 8. The MAE and 3D-SSIM plots suggested that the hemispherical array outperformed the other arrays in the outer cubic shells (cubic shells 7–10) of object space. This, however, was likely due to increased detection of only the lower planes of object space. Packing the transducers more tightly allowed for a greater number of transducers to detect voxels closer to the array surface. The results for array radius optimization are given in Fig 10 and indicated improved system performance with decreasing array radius. This was also due to the inverse relationship between system sensitivity and distance (Eq (8)). At 37.5 mm and 47.5 mm array radius, however, the RMSE and PSNR maps showed the system performed poorly to voxels in the lower and upper planes of object space. The RMSE and PSNR scores gave higher weight to large errors and highlighted regions of poor performance. This trend was also seen in the RMSE and PSNR plots (Fig 10(E) and 10(F)). System performance was proportional to array radius in the outer cubic shells (cubic shells 7–10) of object space and inversely proportional near the center of object space (cubic shells 6–10). Moreover, the system performance difference between array radii decreased in relation to cubic shell 6. The RMSE and PSNR plots showed cubic shell 6 as the intersection point between the array radius curves and appeared to be independent of array radius. The MAE and 3D-SSIM plots did not reveal anything noticeable at cubic shell 6. As array radius decreased, the FoM curves appeared to flatten, signifying a more uniform system performance throughout object space.

### Recommendations for future work

Our findings indicate the FoM from the spatial crosstalk matrix are excellent indicators of performance and other parameters could be considered in order to further improve PA imaging system design. For instance, evaluation of different transducer arrangements and transducer directionality would be two important parameters to increase the field of view. Another important area to improve would be the point source. Techniques from the field of acoustic scale model measurements for generating acoustic point sources could be explored [34]. The point source could also be back-illuminated by optical fibers to account for light fluence in the forward model or calibration in an optical tissue-mimicking medium would improve image reconstruction of structures inside tissues [35]. The proposed scheme could improve these parameters by seeking the optimal FoM scores.

In order to further validate our findings, imaging experiments could also be conducted with the different system designs to compare their respective imaging performance. In addition to IQA metrics, imaging performance can be directly measured using task-based metrics, which help quantify the performance of an observer for a given task. The observer seeks to classify different sections in the image and the task-based metrics provide insight into how successful the observer would be in the detection or classification of a given task. The relationship between the generic FoM and task-based metrics could also be studied.

Compared to our previous work, one advantage of the spatial crosstalk matrix analysis over the SVD metric analysis was the ability to compare unambiguously across different system arrangements due to the availability of a reference standard, i.e. the identity matrix. The identity matrix in this context represented an ideal system response where for each grid point in object space, the system had equal system sensitivity and no aliasing. This is equivalent to making the system shift-invariant. Since this is unlikely in reality, the identity matrix could be weighted in such a way to define a specific PAT system response [21].

Since photoacoustic tomography systems exhibit a frequency-dependent response, another potential method could utilize the wavelet crosstalk matrix. Qi and Huesman demonstrated the application and advantages of the wavelet crosstalk matrix to assess shift-variant imaging systems [21]. The wavelet crosstalk matrix is based on wavelet series expansions and characterizes information from both frequency and spatial domains. Qi and Huesman, however, only introduced the wavelet crosstalk matrix for system assessment in a qualitative manner. Deriving FoM from the wavelet crosstalk matrix may provide greater insight into PAT system performance than the spatial or Fourier crosstalk matrix alone. Nevertheless, an advantage of the spatial crosstalk matrix over the Fourier and wavelet crosstalk matrix is that it can be experimentally measured for a real system, whereas it is extremely difficult to do so for the Fourier and wavelet crosstalk matrix. The system could also be assessed according to its ability to track motion or movement by applying video quality assessment (VQA) techniques to the crosstalk matrix. The 3D-SSIM could be extended into a 4D-SSIM metric, where temporal dynamics make up the fourth dimension, by again extending the work done by others (see Ref. [31]). Other FoM, particularly PVQ metrics, derived from the spatial crosstalk matrix could also be studied.

### System design strategies and considerations

Most PAT staring, sparse array systems have been designed with transducers arranged into rungs, which appears to be sub-optimal based on the FoM results presented in this work. Other transducer arrangements have also been previously implemented. In particular, the 3D PAT system used by Kruger *et al* for breast cancer imaging implemented a spiral pattern to lay out 128 transducers on a hemispherical surface, capturing unique projections in k-space and providing uniform coverage as the array is scanned [7]. Another example includes the hand-held 3D PAT system developed in [13], which densely packed 256 transducers into multiple rungs in a cylindrical cavity, but with the center of the array empty for light delivery. The *uniform sampling arrangement* improved system performance over the rung-based arrangement for the given constraints in this study and represents a simple way to enhance our current system. At the same time, it is important to further compare the performance of the *uniform sampling arrangement* with other previously implemented transducer arrangements.

System performance seemed to be independent of array angular coverage in the elevation direction for Φ > 0°. The MAE and 3D-SSIM measures for the hemispherical array scored better than the other arrays in the outer regions of object space, but, as mentioned above, this was likely due to improved detection for the lower xy-planes in object space. Nevertheless, increasing the angular coverage of the array also increased the sampling over a larger solid angle and provided for a wider distribution of viewing angles. Therefore, either more transducers could be mounted on the bowl or the array radius could be reduced. The optimal array radius size would be determined by the dimensions of the desired object space. For the experimental PAT system, the results suggested that the size of object space should be restricted to a cube with dimensions of 1.2 cm x 1.2 cm x 1.2 cm, corresponding to cube contour 5 (Fig 10(E) and 10(F)). The FoM indicated that an object space of 2 cm x 2 cm x 2 cm would require a larger array radius (around double the current radius) or that the angular acceptance of the transducers would need to be widened. The size of the array should be increased such that every grid point is detected by each transducer and transducers are positioned as close as possible to object space. A recent approach shown capable of improving imaging performance with a limited number of views was done by treating backscatterers (e.g. steel rods placed behind the object) as virtual transducers [36,37]. The backscattered acoustic waves contain the backside information (i.e. regions not within view of the array) of an absorbing object and direct these waves to the transducers. Although the work done in [36,37] utilized raster scanning, the principle could be extended to staring arrays and the spatial crosstalk matrix FoM could be used to optimize the positions of the virtual transducers.

## Conclusion

A PAT system consisting of a 96-element near-spherical array was built and PAT sampling characteristics were used to validate the spatial crosstalk matrix as a tool for quantitative 3D PAT system analysis. Figures-of-merit mirroring IQA metrics were derived from the spatial crosstalk matrix. The FoM used in this work were RMSE, PSNR, MAE and 3D-SSIM3D-SSIM. The response of the FoM were studied as a function of spatial and temporal sampling rate. Increasing spatial and temporal sampling rates (to the Nyquist rate) is well-known to result in improved PAT system performance. The response of the FoM matched these trends, suggesting they were useful for characterizing 3D PAT system designs. We have examined array designs with respect to angular coverage and the FoM were utilized to assess the effect of transducer arrangement, array angular coverage, and array radius on a simulated 129-element spherical transducer array. A uniform sampling arrangement was shown to outperform the rung-based arrangement. Furthermore, we found the FoM to be subtly dependent on the angular coverage of the array, but significantly dependent on the array radius, which implied a trade-off between system performance and field of view. The use of the spatial crosstalk matrix method could be extended with the wavelet crosstalk matrix, which has been shown to be more effective at characterizing shift-variant systems, allowing for more thorough system design analysis of 3D PAT systems.
